# Tumor Vasculature as an Emerging Pharmacological Target to Promote Anti-Tumor Immunity

**DOI:** 10.3390/ijms24054422

**Published:** 2023-02-23

**Authors:** Hong-Tai Tzeng, Yu-Jie Huang

**Affiliations:** 1 Institute for Translational Research in Biomedicine, Kaohsiung Chang Gung Memorial Hospital, Kaohsiung 83301, Taiwan; 2 Department of Radiation Oncology, Chang Gung Memorial Hospital, Kaohsiung 83301, Taiwan

**Keywords:** endothelial heterogeneity, immune response, immunotherapy, tumor angiogenesis, vasculature normalization

## Abstract

Tumor vasculature abnormality creates a microenvironment that is not suitable for anti-tumor immune response and thereby induces resistance to immunotherapy. Remodeling of dysfunctional tumor blood vessels by anti-angiogenic approaches, known as vascular normalization, reshapes the tumor microenvironment toward an immune-favorable one and improves the effectiveness of immunotherapy. The tumor vasculature serves as a potential pharmacological target with the capacity of promoting an anti-tumor immune response. In this review, the molecular mechanisms involved in tumor vascular microenvironment-modulated immune reactions are summarized. In addition, the evidence of pre-clinical and clinical studies for the combined targeting of pro-angiogenic signaling and immune checkpoint molecules with therapeutic potential are highlighted. The heterogeneity of endothelial cells in tumors that regulate tissue-specific immune responses is also discussed. The crosstalk between tumor endothelial cells and immune cells in individual tissues is postulated to have a unique molecular signature and may be considered as a potential target for the development of new immunotherapeutic approaches.

## 1. Introduction

Tumor vasculature is characterized by an abnormal vessel structure with impaired perfusion and is caused by an imbalance between pro- and anti-angiogenic signaling within tumors [1]. An abnormal vessel structure results in inefficient oxygen and nutrient supply to tumor cells and metabolic waste removal. This leads to hypoxia and acidosis in the tumor microenvironment (TME), which supports tumor progression, and the high permeability of vessels allows tumor metastasis to distant organs [2]. In addition, the malfunction of tumor vessels suppresses the recruitment, adhesion, and activity of immune cells and induces immunotherapy resistance [3].

Vascular endothelial growth factor (VEGF) plays a significant role in tumor angiogenesis, and as such, there has been a focus on the development of VEGF pathway-targeted therapeutics with the aim of tumor angiogenesis inhibition by tumor nutrient deprivation [4]. Patients with certain cancer types do not respond to anti-VEGF monotherapy; however, when combined with chemotherapy, they showed improved clinical outcomes [5,6]. In recent years, vascular-targeting strategies to normalize tumor vessel phenotypes were shown to enhance immunotherapy efficacy, suggesting that tumor blood vessels possess an immunomodulatory effect [3]. Using emerging single-cell analysis, it was shown that endothelial cells (ECs) across tissues display unique molecular signatures, and the interplay between tumor ECs and immune cells drives the tissue-specific immunomodulatory roles of ECs [7]. This provides the molecular basis for combining tumor EC targeting and immune checkpoint blockade (ICB) for cancer treatment. This review highlights the potential therapeutic strategy of pharmacologically targeting a dysfunctional tumor vasculature for shaping an anti-tumor immune microenvironment. The mechanisms involved in vascular normalization are also discussed.

## 2. Angiogenesis in the TME

### 2.1. VEGF-Dependent Angiogenesis

Tumor angiogenesis has been implicated in tumor growth and immunosuppression, as well as treatment response [8]. Under normal physiological conditions, vascularization is regulated by the balance between pro- and anti-angiogenic factors. However, an imbalance between these factors promoting a pro-angiogenic state occurs in the TME. High levels of pro-angiogenic factors, such as VEGF, basic fibroblast growth factor (bFGF), platelet-derived growth factor (PDGF), and angiopoietin (Ang), results in abnormal vascular structure formation in the tumor bed and stimulates nutrient and oxygen supply to tumor cells, thereby promoting tumor progression [9,10]. The members of the VEGF family, which includes VEGF-A [11], VEGF-B [12], VEGF-C [13], VEGF-D [14], and placental growth factor (PLGF) [15], represent the most well-defined pro-angiogenic factors known to promote tumor angiogenesis. These factors form biologically active ligands via homo- or heterodimers and interact with their cognate receptors, including VEGFR-1, VEGFR-2, and VEGFR-3 [16]. VEGF-A (also known as VEGF) overexpression enhances vasculature permeability and results in disordered and dysfunctional blood vessels [17]. Signal transduction is initiated by VEGF-A binding to its primary receptor, VEGFR2, on the surface of endothelial cells. Activated VEGFR2 phosphorylates and activates phosphotidylinositol-3 kinase (PI3K)/Akt, leading to increased calcium levels and endothelial NO synthase (eNOS) activity. Therefore, NO production is increased and promotes vessel permeability [18,19]. In murine models, it has been shown that VEGF signaling inhibitors target VEGF-mediated pathways and suppress tumor proliferation and motility [20,21]. 

### 2.2. Anti-Angiogenesis by Thrombospondin-1 (TSP-1)

Several negative angiogenic factors have been identified, including thrombospondin-1 (TSP-1) [22], tissue inhibitors of metalloproteinases (TIMPs) [23], endostatin [24], and angiostatin [25]. These factors can target specific angiogenic pathways and regulate tumor progression. For example, TSP-1 is a potent inhibitor of EC proliferation and survival [26,27]. TSP-1 inhibits vascular cell angiogenesis through the negative regulation of NO biosynthesis and NO-mediated signaling [28]. TSP-1 overexpression has been shown to suppress tumor growth and is associated with a decrease in vascular density [29]. These results indicate that restoring pro- and anti-angiogenic balance may reshape tumor vasculature and delay tumor progression.

## 3. Tumor Vascular Networks Regulate Anti-Tumor Activity

As a growing tumor needs nutrients to meet its demand, cancer cells promote angiogenesis in favor of angiogenic stimulation by the release of pro-angiogenic factors to stimulate new vessel growth. In addition to directly supporting tumor growth, abnormal tumor vascular networks also regulate the tumor immune microenvironment by shaping a hypoxic, acidic, and oxidative TME to facilitate tumor progression.

### 3.1. Hypoxia

Decreased local blood flow to the TME leads to hypoxia and inhibits leukocyte migration. In fact, the hypoxia-inducible transcription factors (HIFs), including HIF-1α, HIF-2α, and HIF-3α [30] mediate signaling pathways that affect several immune cell types and create an immunosuppressive microenvironment. HIF-1α has been shown to promote the differentiation of myeloid-derived suppressor cells at the tumor site toward tumor-associated macrophages (TAMs) to support tumor progression [31]. In addition, in a murine model of breast cancer, it was shown that the ablation of macrophage HIF-1α diminished macrophage-dependent T cell suppression and led to reduced tumor growth [32]. In addition, it was shown that HIF-1α negatively regulates T cell receptor (TCR) signaling to dampen the activity of effector T cells [33]. A recent report has shown that HIF-1α induction by a hypoxic TME maintains regulatory T (Treg) cell fitness via the deubiquitination module. Targeting the ubiquitination process can regulate Treg cell production and consequently promote anti-tumor immunity [34]. HIF-1α upregulates the expression of CD39 and CD73, resulting in the recruitment of plasmacytoid dendritic cells and induces cytotoxic CD8^+^ T cells suppression by Treg cells [35]. Targeting the HIF-1α-CD73 axis effectively facilitates T cell-mediated anti-tumor immune responses and improves ICB response [36]. These results suggest that alleviating tumor hypoxia through blood vessel normalization could alter the tumor-infiltrating immune composition and modulate the anti-tumor immune response.

### 3.2. Cancer Acidity

A typical characteristic associated with abnormal tumor vessels is an acidic TME. Dysregulated energy metabolism results in the accumulation of lactate and a decrease in glucose concentration, resulting in tumor acidification [37]. Regions of acidity and hypoxia typically overlap [38], suggesting that tumor vasculature-induced hypoxia is involved in acidosis in the tumor mass. The low perfusion capacity of tumor vessels restricts oxygen delivery and the removal of acidic wastes, as well as increases metabolic demands in cancer cells, leading to the production and accumulation of acidic metabolites [39]. Several studies have investigated the suppression of anti-tumor immune responses in an acidic TME. In murine models of metastatic breast cancer, the pH was adjusted by the administration of bicarbonate, and tumor metastasis was reduced [40]. In addition, it was shown that the consistent neutralization of tumor acidity inhibited tumor progression and metastasis in prostate and pancreatic cancer animal models [41]. Furthermore, it has been reported that acidic conditions induce anergy in both human and mouse tumor-infiltrating T cells. Restoring the low pH to a physiological value reactivates T cells with functions against tumors [42] and enhances their infiltration to improve the therapeutic efficacy of immunotherapy [43]. These results suggest that an acidic TME limits the effector functions of tumor-specific T cells. In tumors, lactate is one of the major metabolites produced by aerobic glycolysis, and the effects of lactic acid on immune cells regulates tumor progression. High levels of lactic acid impair the production of IL-2, IFN-γ, perforin, and granzyme by cytotoxic T lymphocytes (CTLs) [44]. Accordingly, in patients with melanoma, an inverse correlation between the expression of lactate dehydrogenase, an enzyme responsible for catalyzing the reversible interconversion of pyruvate and lactate, and T cell activation markers was observed, suggesting that lactic acid acts as a T cell inhibitor, thereby suppressing the anti-tumor activity of T cells [45]. In addition, lactate supports tumor growth through HIF-1α activation and resultant TAMs polarization toward the pro-tumoral M2 phenotype [46]. Recently, the transcription factor nuclear factor (erythroid-derived 2)-like 2 (Nrf2) was found to be induced by tumor-derived lactate, and it is also implicated in macrophage M2 type polarization [47]. In contrast, an acidic TME is favorable for the recruitment and development of pro-tumoral myeloid cells. Acidic conditions promote the interaction of P- or L-selectin with its ligand P-selectin glycoprotein ligand-1 (PSGL-1) to promote neutrophil adhesion to the vascular endothelium [48]. Additionally, acidosis induces neutrophil activation through the PI3K/Akt and ERK pathways [49].

### 3.3. Reactive Oxygen Species (ROS)

Low oxygen supply due to a lack of regular tumor vasculature creates a hypoxic TME. It has been shown that hypoxia increases ROS production mainly through tumor mitochondria, and the mitochondrial ROS can also stabilize HIF and activate the HIF-VEGF axis under hypoxia to promote angiogenesis, thereby fostering a TME with oxidative stress [50]. Emerging evidence has shown that ROS acts as a critical regulator of anti-tumor immune functions. For example, the production of free oxygen radicals in the TME could modify cysteine residues in proteins post-translationally, leading to an alteration of antigenicity and alleviating the anti-tumor immunity of T cells [51,52]. Moreover, sustained ROS exposure dampens T cell-mediated inflammation and activation [53]. Additionally, the downregulation of ROS by the inhibition of NOX2, a NADPH oxidase responsible for producing ROS, enhanced IFN-γ secretion by natural killer (NK) cells and suppressed lung metastasis of melanoma [54], suggesting an immunosuppressive role of ROS in NK cell-mediated tumor cytotoxicity. Conversely, ROS promotes macrophage differentiation toward the pro-tumoral M2 phenotype and is critical for their pro-angiogenic and immunosuppressive functions [55]. Indeed, the reduction of ROS by deletion of NADPH oxidases impaired macrophage differentiation from monocytes and their M2 subtype polarization [56]. A mitochondrial chaperon protein, Lon, has been shown to induce the production of ROS-dependent inflammatory cytokines, including IL-6, TGF-β, IL-13, and VEGF-A, and creates a microenvironment that promotes angiogenesis and M2 macrophage polarization [57]. Recently, ROS in the TME has been reported to induce apoptosis of Treg cells due to their vulnerability to oxidative stress. Subsequently, CD39 and CD73 from apoptotic Tregs converted ATP to adenosine to suppress anti-tumor immunity and induced resistance to anti-programmed death-ligand 1 (PD-L1)-mediated immunotherapy [58]. Collectively, ROS in the TME could promote immune suppression for tumor progression, and a reduction of oxidative stress in the TME may reshape the tumor immune microenvironment.

## 4. Interactions between Tumor-Infiltrating Leukocytes and Endothelium

The chaotic tumor vasculature, which consists of vessels with abnormal morphology and high permeability, indicates dysregulated pathways governing vessel formation. This also compromises vascular function through an alteration of the interplay between endothelium and immune cells via multiple mechanisms and leads to tumor cells evading immune surveillance.

### 4.1. Endothelial Cell-Triggered Immune Response

**Figure 1:**
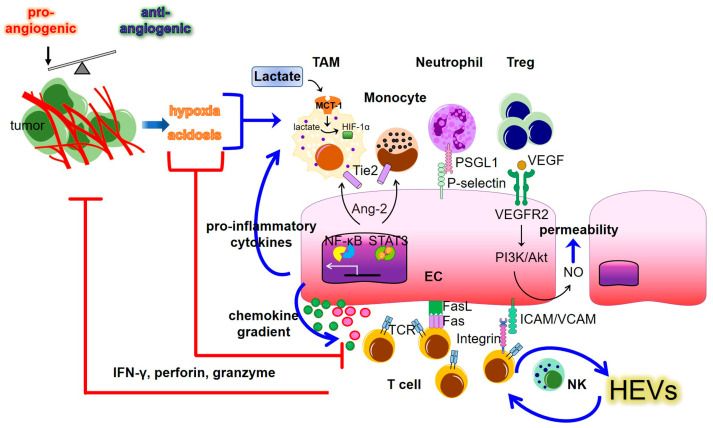
Interactions between tumor endothelial cells (ECs) and immune populations to create the tumor immune microenvironment. Tumor blood vessel abnormalities results from an imbalance between pro- and anti-angiogenic signaling, which in turn results in a hypoxic and acidic TME. The hypoxia and acidosis regulate the immune response through pro-tumoral and anti-tumoral arms. The activation of endothelial cells can facilitate or inhibit tumor immunity through multiple pathways, including contact-dependent and soluble factor-mediated pathways. The blue arrows depict upregulation. The red lines show suppressive effects. MCT: Monocarboxylate transporter; HIF: Hypoxia-inducible factor; TAM: Tumor-associated macrophage; Treg: regulatory T cell; NK: natural killer; Ang-2: Angiopoietin 2; PSGL1: P-selectin glycoprotein ligand-1; VEGF: Vascular endothelial growth factor; VCAM: vascular cell adhesion protein; ICAM: Intercellular adhesion molecule; STAT3: Signal transducer and activator of transcription 3; NF-κB: Nuclear factor kappa-B; TCR: T cell receptor; HEVs: High endothelial venules; IFN-γ: Interferon-γ.

The recruitment of immune cells from blood to inflamed tissues is dependent on their adhesion to ECs, followed by extravasation and infiltration into tissues. This process requires the expression of adhesion molecules, such as E-selectin, intercellular adhesion molecule-1 (ICAM-1), vascular cell adhesion protein 1 (VCAM-1), and VE-cadherin in ECs [59]. However, the abnormal vasculature in tumors creates a TME with hypoxia, acidosis, and reduced blood perfusion, which limits immune cell infiltration and suppresses the anti-tumor immune response [60,61,62] (Figure 1). The upregulation of epidermal growth factor-like domain 7 (Egfl7) in tumors suppresses the expression of adhesion molecules on ECs, thereby preventing leukocyte entry into the tumor mass [63,64]. In addition, tumor ECs can express PD-L1 and Fas ligand to inhibit T cell activation and induce apoptosis of immune effector cells, respectively, leading to an immunosuppressive TME [65,66]. EC-derived angiopoietin 2 (Ang-2) supports tumor progression by inducing Tie2, the Ang-2 surface receptor, which is involved in the recruitment of tumor-associated macrophages and monocytes [67,68]. These results confirm that Ang-2 inhibition reduces tumor-infiltrating monocytes and angiogenesis, leading to a suppression of tumor growth [69].

Additionally, pro-inflammatory signaling elicited by tumor ECs also plays a role in the regulation of immune cell recruitment. Dysfunctional ECs with gene expression profiles mimicking tumor ECs increased the NF-κB-p65 and pSTAT3 levels in the nucleus, suggesting the induction of pro-inflammatory signaling. Indeed, GM-CSF, IL-8, and IL-6 overexpression with reduced levels of anti-inflammatory cytokines, confirmed the pro-inflammatory phenotype in TME. Dysfunctional EC properties, including increased proliferation, permeability, and monocyte adhesion, have also been observed in this phenotype [70]. A recent study has shown that the expression of the innate receptor Toll-like receptor 2 (TLR2), which induces the production of a panel of pro-inflammatory cytokine in endothelial cells, promotes pro-tumorigenic immune cell recruitment via cell surface adhesion molecule (P-selectin, E-selectin, ICAM-1, and VCAM-1) upregulation [71]. 

Although abnormal tumor vasculature inhibits leukocyte recruitment, tumor EC malfunction preferentially attracts and recruits pro-tumoral immune populations. For example, Clever-1, a scavenger and adhesion receptor, regulates monocytes/macrophages and Treg tumor infiltration when expressed in tumor ECs [72]. Clever-1 deletion in ECs or the administration of an anti-Clever-1 antibody decreased immunosuppressive immune infiltrates in the tumor mass, including monocytes/macrophages and Treg cells, but not T lymphocytes [72]. Similarly, an intracellular molecule, apoptosis signal-regulating kinase 1 (ASK-1), activates ECs and promotes tumor infiltration of macrophages but does not affect T lymphocyte recruitment. ASK-1 inhibition hinders macrophage entry into the tumor bed and leads to tumor growth retardation [73]. In addition, in murine models, endothelial deletion of SHP-2, an intracellular phosphatase responsible for ASK-1 stabilization (which is required to maintain EC activation) reduces microvascular density and normalizes tumor vasculature, leading to tumor growth inhibition [74]. Interestingly, ASK-1 inhibition reduces TLR-4-mediated inflammatory responses, including IL-6, IL-8, soluble VCAM, and G-CSF secretion from ECs [75], suggesting that EC-produced soluble immune mediators are involved in the regulating accessibility of immune cell types to tumor regions. Indeed, EC-derived chemotaxis also plays a crucial role in immune cell recruitment. Expression of chemokine (C-X3-C motif) ligand 1 (CX3CL1) by ECs specifically mediates the CX3CL1 receptor (CX3CR1)-expressing monocyte recruitment into tumors [76]. 

In contrast to EC-mediated tumor recruitment of pro-tumoral immune cell types, the activation of ECs also induces anti-tumor T lymphocyte entry into tumors. EC stimulation with the agonist of innate immune sensor melanoma differentiation-associated protein 5 (MDA5) induced the production of type I interferon and T cell-recruiting chemokines, such as CXCL9 and CXCL10, leading to increased tumor-infiltrating CD8^+^ T cells and an augmented anti-tumor immune response [77]. In addition, neutral sphingomyelinase 2 (nSMase2)-dependent EC activation has been shown to express chemokines, CX3CL1 and CXCL10, as well as the adhesion molecule VCAM1, to promote T cell migration into the tumor bed. Inhibition of nSMase2 decreased the expression of VCAM1, CX3CL1, and CXCL10 by ECs and reduced T cell tumor infiltration [78]. Conversely, tumor-infiltrating lymphocytes also contribute to the regulation of vascular normalization, as CD8^+^ T cell depletion hinders tumor blood vessel remodeling [79]. These results imply that the interaction between ECs and lymphocytes regulates tumor vascular networks. Collectively, these results propose that a variety of EC activation pathways, in response to various stimuli, may shape the immune microenvironment by mediating the recruitment of specific immune cell types into the tumor bed.

### 4.2. High Endothelial Venules (HEVs)

HEVs are specialized postcapillary venules that express sulfated sialomucins, which interact with L-Selectin/CD62L-expressed naïve lymphocytes, leading to migration into the lymph nodes [80]. HEVs are found in various types of cancer, and their enrichment in tumor regions is associated with better prognosis [81,82,83]. Recently, the molecular signature of HEVs revealed that upregulated HEV genes, such as MEOX2 and TSPAN7, were associated with improved survival rate and increased tumor-infiltrating T and B cells in advanced breast cancer [84]. It has also been reported that tumor-associated HEVs are the major sites for lymphocyte migration into tumors, and the HEVs density is positively correlated with tumor-infiltrating stem-like CD8^+^ T cells. More importantly, tumor-associated HEVs are predictive factors for a better response in patients with metastatic melanoma on combined treatment with anti-PD-1 and anti-CTLA-4 immunotherapy [85]. Furthermore, tumor necrosis factor-mediated R-Ras upregulation in HEVs is responsible for naïve T cell migration to the lymph nodes. Inactivation of endothelial R-Ras reduces the development of anti-tumor activity in antigen-specific T cells [86]. Notably, combinatorial treatment with anti-VEGF therapy and anti-PD-L1 immunotherapy induces HEV formation and tumor infiltration of T cells, resulting in an enhanced anti-tumor immune response [87]. Consistent with these results, induction of HEV clusters, by targeting the vascular LIGHT protein, stimulates the migration of effector T cells into tumor beds and sensitizes refractory tumors to immunotherapy [88,89]. Recently, a study has demonstrated that induction and maintenance of intratumoral HEVs require sustained signaling (e.g., IFN-γ-mediated pathways) from CD8^+^ T and NK cells, suggesting a mutual regulation of tumor vessels and anti-tumor immune effector cells [90]. Thus, specialized vascular networks provide an emerging therapeutic target with immunomodulatory effects for regulating lymphocyte migration into tumors.

### 4.3. Heterogeneity of ECs

Since ECs are the interior lining of blood vessels, ECs are key components of the vascular system. Although these cells share some common features, they do not have homogeneous phenotypes across tissues or even within a single tissue [91]. The functional heterogeneity of ECs has been revealed by the development of single-cell analysis technology in recent years [92]. ECs from different tissues express gene signatures that are enriched in distinct biological processes. For example, highly expressed EC gene set in the liver and spleen have been implicated in scavenging and immunoregulation [92,93]. ECs from the colon and small intestine express genes enriched for VEGF signaling, EC migration, and the maintenance of vascular barrier integrity [92,94]. ECs also express unique organ-specific markers. Adhesion molecules responsible for immune cell recruitment, such as VCAM-1 and scavenger proteins, are highly expressed in periportal liver sinusoidal ECs (LSECs) and midlobular LSECs, respectively, whereas genes involved in angiogenesis, such as VEGF-A and TSP-1, are expressed predominantly in pancreatic ECs [95]. Therefore, the functional heterogeneity of ECs may involve tissue-specific molecular mechanisms to react with the surrounding environment. 

ECs in tumors leverage distinct mechanisms to regulate tumor progression by modulating the immune microenvironment. LSECs have been reported to suppress the naïve CD8^+^ T cell differentiation into mature effector T cells with tumoricidal activity, thereby suppressing anti-tumor immunity [96]. Expression of the EC-specific protein, PLVAP in LSECs, accelerates tumor recruitment of FOLR2-expressed TAMs to create an immunosuppressive niche in hepatocellular carcinoma [97]. In lung small cell lung cancer, the expression of genes involved in chemotaxis, including CCL2, CCL18, IL-6, adhesion molecule ICAM-1, and antigen presenting complex MHCI/II in tumor ECs are down-regulated [98]. In addition, ECs in lung cancer express high levels of Fas ligand to trigger the apoptotic pathway in tumor-infiltrating cytotoxic T cells [66], but also the inhibitory molecule PD-L1 to suppress the activation of effector T cells, indicating their immunosuppressive roles [7]. Collectively, the gene expression profiles involved in immunomodulatory pathways in tumor ECs differ across organs, suggesting that tumor ECs in different organs exploit diverse molecular mechanisms to shape the tumor immune microenvironment.

## 5. Normalization of Tumor Vasculature Improves Response to Immunotherapy

Given that abnormal vascular networks in tumor masses play a critical role in regulating the anti-tumor immune response, targeting tumor blood vessels to establish a favorable microenvironment for the accessibility of anti-tumor effector immune cells reveals an approach to improve the therapeutic efficacy of immunotherapy.

### 5.1. Immune Checkpoint Blockade (ICB)

The development of a normal vascular structure depends on the balance between angiogenic stimulators (e.g., VEGF) and angiogenic inhibitors such as TSP-1. The imbalance resulting from excessive stimulators in the TME leads to the development of an abnormal and dysfunctional vasculature structure. Therefore, restoring the balance in the angiogenic process may restore the vessel structure in tumors. Since abnormal tumor vasculature results in the inhibition of immune cell infiltration, which leads to immunotherapy resistance, the normalization of tumor vascular networks to promote effector immune cell entry into tumors is a potential strategy to improve the efficacy of ICB [99]. Given that VEGF-mediated signaling pathways are the major drivers of tumor angiogenesis, anti-angiogenic therapy targeting VEGF-dependent signaling to normalize tumor vasculature represents an emerging therapeutic strategy [100]. High levels of VEGF increase the permeability of tumor vessels and create disordered, leaky vasculature in tumors [17]. Inhibition of VEGF signaling, using specific monoclonal antibodies or small molecules, restores functional tumor vessels and reverses the hypoxic TME to promote immune cell infiltration [101,102]. More importantly, the increased recruitment of IFN-γ-producing T cells, due to tumor vasculature remodeling, further promotes the expression of adhesion molecules on ECs for effector immune cell infiltration. Thus, this forms a positive regulatory loop between vascular normalization and T cell-dependent anti-tumor immune response [79]. However, the use of VEGF inhibitors for treating cancer is often associated with adverse effects [103]. Molecules involved in VEGF-mediated pathways should be considered as potential therapeutic targets. In murine models, CD93, a receptor expressed on ECs, was explored as a therapeutic target and identified as a downstream marker linked to the efficacy of anti-VEGF therapy. Interaction with its ligand, insulin-like growth factor binding protein 7 (IGFBP7), contributes to the formation of a dysfunctional tumor vessel structure. Targeting the CD93-IGFBP7 axis, using monoclonal antibodies reverses the properties associated with the abnormal tumor vasculature structure, including hypoxia, vessel leakage, and immunosuppression, leading to an improved ICB response [104]. In addition, a recent report showed that the endothelial expression of the innate immune sensor stimulator of interferon gene (STING) responsible for mediating cytosolic DNA-induced inflammatory response [105], was correlated with the accumulation of tumor-infiltrating T cells in patient samples, and was expected to be a valuable prognostic marker. Agonists that stimulate STING-mediated signaling reprogrammed abnormal tumor vasculature toward a normalized vascular structure and displayed synergistically therapeutic efficacy when combined with of VEGFR2 and PD-1 blocking [106]. However, STING activation can upregulate VEGF expression in the dysfunctional retinal pigment epithelium and result in pathological angiogenesis in retinal vascular diseases [107], suggesting that environmental factors also play crucial roles in VEGF-dependent pathological angiogenesis.

### 5.2. CAR-T Efficacy

Chimeric antigen receptor (CAR)-based immunotherapy may be critical in overcoming the barriers derived from aberrant tumor vasculature to improve T cell tumor infiltration. CAR contains an extracellular domain from a single-chain variable fragment (scFv) antibody for antigen recognition linked to a cytoplasmic signaling domain, which consists of a costimulatory fragment to activate T cells when engaged with specific tumor antigens [108]. Thus, the accessibility of engineered CAR-T cells to the tumor mass determines the efficacy of this type of immunotherapy. A study showed that the use of engineered T cells with CARs, and scFv from antibodies targeting VEGFR2, showed durable and increased T cells entry into tumors, a phenomenon correlating with the suppression of tumor progression [109]. Another endothelial antigen targeted by CAR-T is the tumor endothelial marker (TEM) 8. Treatment with TEM8-specific CAR-T cells, in a murine model of triple-negative breast cancer, blocked tumor neovascularization and induced tumor regression [110]. Additionally, CAR-T targeting another endothelial marker, C-type lectin domain-containing 14A (CLEC14A), has been shown to reduce vascular density and diminish tumor burden [111]. To improve the efficacy of CAR-T, the simultaneous targeting of VEGFR2 and tumor antigen by CAR-T improves anti-tumor activity by increasing antigen-specific T cell infiltration [112]. Thus, the use of CAR-T targeting the tumor endothelium directly not only rebalances the actions between pro- and anti-angiogenic factors for vessel normalization, but also enhances the tumor accessibility of T cells, thereby representing a promising strategy for cancer treatment. Selected animal studies related to the combination treatment of vascular normalization and immunotherapy are summarized in Table 1.

## 6. Resistance to Anti-VEGF Therapies

**Figure 2:**
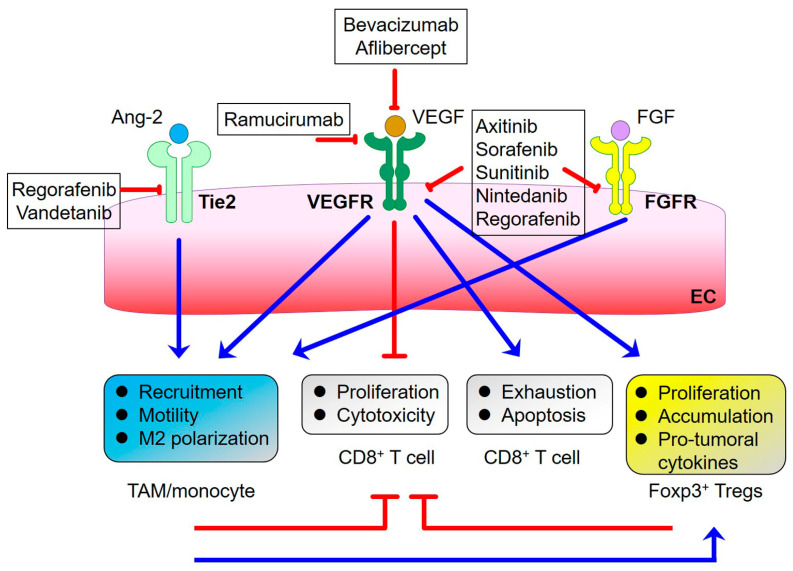
Immunomodulation of pro-angiogenic pathways and the approved drugs that target them. Triggering of VEGFR (mainly VEGFR2) pathways by the cognate ligands promotes CD8^+^ T cell exhaustion and apoptosis and inhibits their anti-tumor activity. Additionally, the immunosuppressive functions of TAMs and Foxp3^+^ Tregs are also induced. Tie2- and FGFR-mediated pathways activate pro-tumoral activity by recruiting TAMs/monocytes, which can further promote Treg-dependent tumor-supporting activity. The blue arrows depict activation. The red lines show inhibition. Bevacizumab and Aflibercept are VEGF inhibitory agents. Ramucirumab is the monoclonal antibody targeting VEGFR2. Axitinib, Sorafenib, Sunitinib, Nintedanib, and Regorafenib are tyrosine kinase inhibitors. Regorafenib and vandetanib are multi-kinase inhibitors that can inhibit Tie2-dependent signaling. TAM: tumor-associated macrophage; Treg: regulatory T cell; Ang-2: angiopoietin 2; VEGF: vascular endothelial growth factor; FGF: fibroblast growth factor; VEGFR: VEGF receptor; FGFR: FGF receptor; EC: endothelial cell.

Although VEGF blockade exhibits promising anti-cancer therapeutic efficacy, by normalizing tumor vasculature, the optimal time and dosage of anti-VEGF treatments have been proposed to be the determinants for effective vessel normalization [2,117]. During the period of normalization window, increased tumor oxygenation and drug delivery, as well as improved efficacy of combined therapy with VEGF targeting and cytotoxic drugs, were observed [4]. Conversely, an administration of high doses of VEGF inhibitors resulted in toxicity in normal tissues and an increase in tumor hypoxia due to the degeneration of tumor blood vessels, thereby stimulating tumor progression [118]. Additionally, immunosuppression-regulated resistance to VEGF targeting implies the contribution of other regulators to tumor promotion via immunomodulation (Figure 2). It has been shown, in a murine model of gliomas, that anti-VEGFR2 therapy induces Ang-2 expression, which inhibits vessel normalization by VEGFR2 inhibition and adversely affects survival [119]. Ang-2 overexpression due to VEGFR2 blockade increases tumor-associated macrophages (TAMs) infiltration and leads to therapeutic failure, suggesting the reshaping of the tumor immune microenvironment toward pro-tumoral phenotypes. Indeed, dual inhibition of Ang-2 and VEGF in a murine glioblastoma model with highly abnormal tumor vessels decreased vessel density and reprogrammed TAMs toward an anti-tumor phenotype and improved the therapeutic efficacy of anti-VEGF monotherapy [120]. More recently, the combination of a bispecific nanobody, targeting VEGF and Ang-2, with PD-1 blockade, showed profound anti-tumor activity in a lung cancer murine model. This further demonstrated increased tumor infiltration of anti-tumor immune cells, via inhibition of pro-angiogenic signaling [121]. However, in an autochthonous lung tumor model, a PD-1 or PD-L1 blockade was not beneficial to the dual inhibition of VEGF and Ang-2. This was due to the increased tumor-infiltrating PD-1^+^ Treg cells, which were induced by anti-angiogenic treatments. Notably, the inhibition of TAMs reduced Treg cell infiltration [122], suggesting that a blockade of angiogenic molecules recruits TAMs to alleviate anti-tumor immunity. Other pro-angiogenic molecules have also demonstrated involvement in anti-VEGF therapy resistance. Fibroblast growth factor 2 (FGF-2)-dependent signaling pathways are required for resistance to anti-VEGF therapy. FGFR inactivation decreases tumor vessel density and restores anti-VEGF therapy efficacy [123,124]. Inhibition of hepatocyte growth factor (HGF)-mediated signaling prevents aberrant vascular morphology and ameliorates VEGFR inhibitor resistance in non-small cell lung cancer [125]. Interestingly, the administration of FGF-2 and HGF can induce vessel growth and recruit M2-like macrophages in tissue [126], suggesting an immunosuppressive microenvironment created by these pro-angiogenic factors. Therefore, the anti-angiogenic cocktail may be required for a better treatment response. In fact, there have been developments in the targeting of multiple kinases involved in pro-angiogenic pathways, such as VEGFR, FGFR, PDGFR, and the Tie2 angiopoietin receptor, which blocks the spectrum of angiogenic signaling. For example, the exposure of the multi-target tyrosine kinase inhibitor, sorafenib, which inhibits VEGFR1-3 and PDGFR, sensitized hepatocellular carcinoma to anti-PD-1 therapy. This indicated the presence of normalized tumor vessels and an increased proportion of tumor-infiltrating T cells [127]. In fact, the administration of sorafenib amplified vascular normalization, promoted tumor reoxygenation, and reversed the immunosuppressive tumor microenvironment [128]. In addition, in a murine model of breast cancer, the production of IL-6 from adipocytes and tumor-infiltrating myeloid cells, such as macrophages, has been demonstrated to contribute, at least partly, to refractoriness to anti-VEGF treatment. Inhibition of IL-6 normalizes the tumor vasculature and reduces the recruitment of immunosuppressive cells, such as Treg cells to abrogate resistance to anti-VEGF therapy [124]. Tumor-infiltrating CD11b^+^Gr1^+^ myeloid cells with high gene expression, implicated in their mobilization and recruitment, play an important role in the anti-VEGF treatment resistance [129]. Collectively, these results suggest that though vessel-targeting therapies reduce tumor vascular density and normalize tumor blood vessels, immune profiling analysis in the reshaped tumor immune microenvironment may provide opportunities for the development of more effective treatment strategies when combined with vessel-targeting and immunotherapy.

## 7. Clinical Implications of Targeting Tumor Vasculature

The immunosuppressive effects of tumor vascular networks provide a rationale for combining inhibitors targeting the tumor vasculature with immune checkpoint blockade for cancer treatment. Bevacizumab, a monoclonal antibody against VEGF as a cancer treatment, increases the dendritic cell (DC) population, improving their T cell stimulatory capacity [130]. A recent study revealed that cancer patients with a gene set enriched in the DC population have an improved response to immune checkpoint inhibitors [131]. In addition, in patients with advanced gastric cancer, targeting VEGFR2 with ramucirumab induces CD8^+^ T cell infiltration and simultaneously decreases tumor-infiltrating Tregs [132]. These results suggest the reshaping of the TME as the results of tumor vasculature remodeling. A combination of VEGF inhibitors and anti-immune checkpoint therapy confers improved clinical outcomes for several cancer types [133,134,135,136]. Lenvatinib, a multikinase inhibitor targeting VEGFR1, VEGFR2, and VEGFR3, and receptor tyrosine kinases, has been used in combination with the anti-PD-1 antibody pembrolizumab in patients with advanced endometrial cancer in a clinical trial. Compared to each monotherapy, an increase in anti-tumor activity without serious adverse events was observed [133]. Patients with advanced hepatocellular carcinoma (HCC) have a longer overall and progression-free survival when treated with a combination of bevacizumab and PD-L1 blocking antibody atezolizumab, compared to those treated with sorafenib [137]. In accordance with the efficacy of dual blockade of VEGF and immune checkpoints, tissues from metastatic renal cell carcinoma patients treated with bevacizumab and atezolizumab show increased intratumoral CD8^+^ T cells and markers related to activated immune responses, such as MHC molecules, effector T cells, and CX3CL1 chemokine [138]. Clinical studies and ongoing trials targeting tumor vessels and immunosuppressive pathways are summarized in Table 2.

## 8. Concluding Remarks

The tumor vasculature comprises abnormal and dysfunctional blood vessel networks that results in an immunosuppressive microenvironment that supports tumor progression. The dual blockade of angiogenic activation and immune checkpoint-mediated inhibitory pathways has led to remarkable advances in the treatment of cancers. However, the potency of combination therapy may be hindered due to the heterogeneity of endothelial cells across and within tissues. Therefore, clinical manipulation of organ-specific ECs by targeting their unique molecular features may inform the development of novel strategies to maximize patient response to immunotherapy. In addition, long-term clinical trials are necessary for the evaluation of the durable clinical benefits of combination therapies for cancer treatment.

## Figures and Tables

**Table 1:** Animal studies of combined treatment of vascular targeting and immunotherapy.

Agents	Target	Cancer Type	Immunomodulation and Response to Combining Immunotherapy
7C10 (anti-CD93 mAb)/2C6 (anti- IGFBP7)	CD93/IGFBP7	pancreatic cancer, melanoma	Intratumoral CD3^+^ T, NK, NKT cells ↑ Foxp3^+^ Treg, MDSC ↓ Tumor regression by combining with anti-PD-1/anti-CTLA-4 mAb Survival ↑ [104]
cGAMP (STING agonist)/DC101 (anti-VEGFR2 mAb)	STING, VEGFR2	LLC, colon cancer	CD8^+^ T, IFN-I ↑ Vessel normalization, anti-tumor immunity ↑ Tumor regression and survival ↑ [106]
B20-4.1.1 (anti-VEGF mAb)	VEGF	small cell lung cancer	PD-1^+^/TIM-3^+^ T cells ↓ by combining anti-VEGF with anti-PD-L1 Improved PFS and OS [113]
Sunitinib (VEGFR, TKI)	VEGFR1, R2, and R3	colorectal cancer	Exhaustion of CD8T ↓ Suppression of tumor growth by combining VEGF-A–VEGFR and PD-1 blockade [114]
DC101 (anti-VEGFR2 mAb)	VEGFR2	colorectal cancer	Restores anti-tumor activity of T cells by combining with anti-PD-1 mAb TAMs ↓ Tumor control ↑ [115]
DC101-CAR-T (scFv from anti-VEGFR2 mAb)	VEGFR2	melanoma, colon carcinoma, fibrosarcoma, renal cancer	Tumor infiltration of CAR-T ↑ Tumor growth ↓ Mice survival ↑ [109]
DC101-CAR-T (scFv from anti-VEGFR2 mAb +TCR against tumor antigens)	VEGFR2/tumor antigens	melanoma	Persistence of Ag-specific T cell infiltration ↑ Tumor-free survival ↑ Tumor growth ↓ [112]
CAR-T (anti-PSMA scFv)	Endothelial PSMA	ovarian cancer	Tumor vascular density ↓ Tumor regression ↑ [116]

↑ indicates increase in cell numbers, frequency or activity; ↓ denotes decrease in cell numbers or populations; IGFBP7: insulin-like growth factor binding protein 7; NK: natural killer; MDSC: myeloid-derived suppressor cells; STING: Stimulator of interferon genes; LLC: Lewis lung carcinoma; IFN-I: type I interferon; PFS: progression-free survival; OS: overall survival; scFv: single-chain variable fragment; Ag: antigen; PSMA: prostate-specific membrane antigen (PSMA); TKI: tyrosine kinase inhibitor; TAMs: tumor-associated macrophages.

**Table 2:** Clinical studies and ongoing trials of the combined treatment of vascular targeting and immunotherapy.

Drug	Target	Cancer Type	Clinical Response
Bevacizumab	VEGF	renal cell carcinoma	Elevated Safety profile Prolonged PFS vs. sunitinib [139]
Atezolizumab	PD-L1		
Axitinib	VEGFR1, R2, and R3	renal-cell carcinoma	Prolonged PFS vs. sunitinib Longer OS than sunitinib Higher objective response rate [134]
Pembrolizumab	PD-1		
Bevacizumab	VEGF	relapsed ovarian cancer	Enhanced activity in the platinum-sensitive group [135]
Nivolumab	PD-1		
Bevacizumab	VEGF	hepatocellular carcinoma	Prolonged PFS vs. sorafenib Longer OS than sorafenib [136]
Atezolizumab	PD-L1		
Bevacizumab	VEGF	renal cell carcinoma	Increase in intratumoral CD8^+^ T, T cell activation markers and CX3CL1 Increase in CX3CR1 on peripheral CD8^+^ T cells Improved antigen-specific T cell migration [138]
Atezolizumab	PD-L1		
Apatinib	VEGFR2	hepatocellular carcinoma	NCT05313282, phase III
Camrelizumab	PD-1		
Anlotinib	VEGFR, FGFR, PDGFR, c-kit	advanced renal cancer	NCT04523272, phase III
TQB2450	PD-L1		
Bevacizumab	VEGF	colorectal cancer	NCT02997228, phase III
Atezolizumab	PD-L1		
HLX04	VEGF	non-small-cell lung	NCT03952403, phase III
HLX10	PD-1		
Bevacizumab	VEGF	hepatocellular carcinoma	NCT05665348, phase III
Atezolizumab/Ipilimumab	PD-L1/CTLA-4		
Bevacizumab	VEGF	ovarian, fallopian tube, or primary peritoneal cancer	NCT02839707, phase II/III
Atezolizumab	PD-L1		
Bevacizumab	VEGF	metastatic colorectal cancer	NCT02997228, phase III
Atezolizumab	PD-L1		
Cabozantinib	VEGFR2, MET, AXL	renal cell carcinoma	NCT03793166, phase III
Nivolumab/ Ipilimumab	PD-1/CTLA-4		
Bevacizumab	VEGF	cutaneous melanoma	NCT01950390, phase II
Ipilimumab	CTLA-4		
Lenvatinib	VEGFR, FGFR, PDGFR, c-kit	hepatocellular carcinoma	NCT04444167, phase I/II
AK104	PD-1/CTLA-4		

PFS: progression-free survival; OS: overall survival.

## Data Availability

Not applicable.
